# Numerical and Experimental Analysis of Horizontal-Axis Wind Turbine Blade Fatigue Life

**DOI:** 10.3390/ma16134804

**Published:** 2023-07-03

**Authors:** Imran Shah, Abdullah Khan, Muhsin Ali, Sana Shahab, Shahid Aziz, Muhammad Adnan Aslam Noon, Javed Ahmad Khan Tipu

**Affiliations:** 1 Department of Mechanical Engineering, International Islamic University (IIUI), Islamabad 44000, Pakistan; adnan.aslam@iiu.edu.pk (M.A.A.N.); javed.tipu@iiu.edu.pk (J.A.K.T.); 2 Department of Mechanical Engineering, National University of Technology (NUTECH), Islamabad 44000, Pakistan; nawababdullah766@gmail.com; 3 Department of Material Engineering, King Abdullah University of Science and Technology, Thuwal 23955-6900, Saudi Arabia; muhsin.ali@kaust.edu.sa; 4 Department of Business Administration, College of Business Administration, Princess Nourah Bint Abdulrahman University, P.O. Box 84428, Riyadh 11671, Saudi Arabia; sshahab@pnu.edu.sa; 5 Department of Mechanical Engineering, Jeju National University, 102 Jejudaehak-ro, Jeju-si 63243, Republic of Korea; shahid@jejunu.ac.kr; 6 Institute of Basic Sciences, Jeju National University, 102 Jejudaehak-ro, Jeju-si 63243, Republic of Korea

**Keywords:** wind turbine blade, fatigue life, goodman model, von Moises stresses

## Abstract

Horizontal-axis wind turbines are the most popular wind machines in operation today. These turbines employ aerodynamic blades that may be oriented either upward or downward. HAWTs are the most common non-conventional source of energy generation. These turbine blades fail mostly due to fatigue, as a large centrifugal force acts on them at high rotational speeds. This study aims to increase a turbine’s service life by improving the turbine blades’ fatigue life. Predicting the fatigue life and the design of the turbine blade considers the maximum wind speed range. SolidWorks, a CAD program, is used to create a wind turbine blade utilizing NACA profile S814. The wind turbine blade’s fatigue life is calculated using Morrow’s equation. A turbine blade will eventually wear out due to several forces operating on it. Ansys software is used to analyze these stresses using the finite element method. The fatigue study of wind turbine blades is described in this research paper. To increase a turbine blade’s fatigue life, this research study focuses on design optimization. Based on the foregoing characteristics, an improved turbine blade design with a longer fatigue life than the original one is intended in this study. The primary fatigue parameters are the length of a chord twist angle and blade length. The experimental data computed with the aid of a fatigue testing machine are also used to validate the numerical results, and it is found that they are very similar to one another. By creating the most effective turbine blades with the longest fatigue life, this research study can be developed further. The most effective turbine blades with the longest fatigue life can be designed to further this research investigation.

## 1. Introduction

The definition of a horizontal-axis turbine is: “Turbines in which the rotor axis is parallel to the wind stream and the wind turns turbine blades around a rotor that spins a generator, producing power.” [1,2]. Horizontal-axis Turbines provide better efficiency and power, and as a result, they are used in large-scale power plants, large-scale wind farms, and also for electricity generation [3]. Because they are in charge of collecting wind energy and subsequently propelling the turbine rotor, blades are the functional part of a turbine blade [4]. Turbine blades extract kinetic energy of wind from the atmosphere and convert it into mechanical energy [5,6,7].

Fatigue failure [8] is defined as the failure that occurs due to the propagation of cracks [9] and repetitive or cyclic stresses [10]. The turbine blade often fails as a result of the start and spread of cracks brought on by fatigue failure [11]. The service life of the turbine blade can be increased by [12] enhancing its fatigue strength. Fatigue strength depends upon the twist angle, chord length, and blade length [13]. Fatigue strength increases by increasing the chord length, and decreases by increasing the blade length [14]. The fatigue life and twist angle have a parabolic relationship; by increasing the twist angle, fatigue life grows tremendously [15].

First-stage blade failure [16] in a Horizontal-axis wind turbine is analyzed where the natural frequencies and vibrations are found in various conditions [17]. Areas where the high stress [17] and temperature [18] cause significant creep growth are traced by using three-dimensional finite stress and thermal analysis, whereas the second stage of HWT blade failure is carried out by performing mechanical examinations of the failed blade.

Numerous other research works employing the finite volume method have examined and assessed the fatigue life of a turbine blade in the literature. Studies that concentrate on extending a turbine’s fatigue life to optimize the turbine blade are, however, scarcely available. Using experimental and numerical methodologies, Wang et al. developed a fracture development life evaluation method for wind turbines that incorporates cycle fatigue loading [18]. They have also conducted some growth tests under high–low combined cycle fatigue loading along with the temperature distributions. Schweitzer et al. [19] explained the fatigue analysis that is performed for the notched linearly varying rotating blade; alternating mass, stress energy stress intensity, and the life of the rotating blade are found. The rain counting method was used for identifying and tracing the damaging events, and one of two methods (Manson or Morrow) was used to incorporate the mean stress effects on fatigue life in a research study by Issier et al. [20], who also performed thermal analysis to find the high-temperature creep life. Nehru et al. [21] performed numerical low-cycle fatigue analysis to predict minimum fatigue life for a blade geometry. This research work sums up the previous contributions of authors. In this paper, a wind turbine blade constructed from a NACA S814 has been investigated experimentally and numerically. The Morrow equation is used to determine the fatigue life. The analysis used in this study also included a method for improving turbine blade geometry to extend the fatigue life of the blades.

The breakdown of turbine blades is a result of fatigue brought on by cyclic or variable load. The lifespan of a turbine blade is approximately 15 to 20 years on average; current research focuses on ways to extend its lifespan. By lengthening a turbine blade’s life by enhancing its fatigue life, this study answers a current demand for research. Since fatigue is the main cause of turbine blade failure, the life of the blade can be extended by extending the fatigue life. The NACA profile S814 has been selected for experimental and numerical investigation. This study’s improved design for a horizontal-axis turbine blade has a longer fatigue life than the original one. The geometry of a blade’s design may change, improving and lengthening fatigue life. Comparing the suggested blade design to the original design, the chord length is longer and the twist angle is higher. These two geometrical features can increase a blade’s fatigue life by decreasing the likelihood of fatigue failure. In these findings, fatigue analysis is carried out experimentally and numerically, and the results are found to be comparable, allowing the numerical model to be validated against the experimental work.

## 2. Design of a Turbine Blade

Turbines with blades that are pointed upward or downward are referred to as horizontal-axis wind turbines. These turbines typically have three or two blades and operate at extremely high speeds. The NACA profile S814 is used in this research study’s turbine blade design on Cad software called SolidWorks for performing numerical and experimental fatigue analysis.

**Figure 1:**
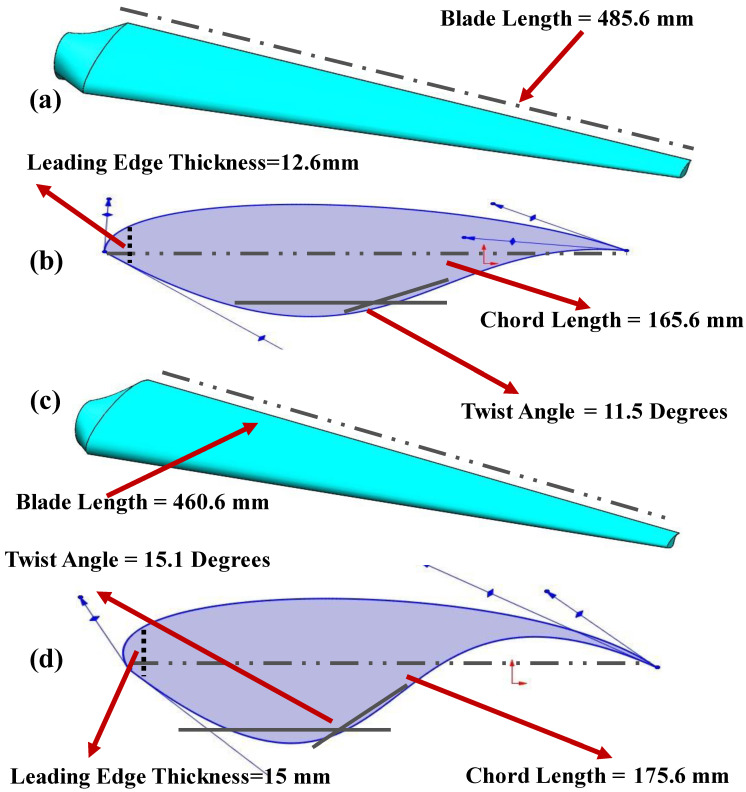
(**a**) Turbine blade’s original airfoil (**b**) A view of a turbine blade’s original shape in three dimensions (**c**) A proposed turbine blade’s airfoil (**d**) A 3D rendering of a turbine blade’s proposed geometry.

These three variables (Figure 1)—chord length, blade length, and leading-edge thickness—determine the fatigue life of a structure. The fatigue life of the turbine blade is increased by properly modifying these parameters. The leading edge of a turbine is where a crack that develops as a result of cyclic stress starts, hence thickening the leading edge slows down crack initiation [22].

## 3. Numerical Simulation Models

When the turbine blade is rotating at the cut-in and cut-out wind speeds, an investigation is conducted. When the turbine blade rotates at the cut-out speed, it experiences the highest stress, and when it rotates at the cut-in speed, the lowest stress. The finite volume method is used to calculate these stresses. Calculations of the stress amplitude (a) and fatigue life of turbine blades can be performed using the obtained stresses. Using Morrow’s equation, the fatigue life of wind turbine blades is estimated.

(1) $$\sigma_{a}=(\sigma_{f}-\sigma_{m})\times {\left(2N_{f}\right)}^{b}$$

$$\sigma_{a}=Stress\;Amplitude$$

$$\sigma_{f}=Fatigue\;Stress$$

$$\sigma_{m}=Mean\;Stress$$

$$N_{f}=Number\;of\;Stress\;Cyles\;leading\;to\;the\;fatigue\;failure\;$$

b=Slope of the line(value varies from 0.04 to 0.15)

According to Morrow, the monotonic yield and ultimate strength of a material cannot adequately represent its fatigue behavior. Morrow believed that the fatigue strength coefficient could never be exceeded by the sum of the stress amplitude and mean stress. The fatigue strength of the material at one reversal is the definition of the fatigue strength coefficient. The fatigue strength coefficient on the mean stress axis and the endurance strength on the amplitude axis are connected by a line with a slope of b.

By optimizing the blade geometry, this research study extends the fatigue life of the wind turbine blades without sacrificing either their fatigue life or their ability to generate power. The optimization procedure is somewhat constrained by the power factor, which plays an important role. The torque created along the blade of the wind turbine is what generates the power. By integrating the total power obtained from the wind turbine blade from the initial zero length to the length of the blade, the major torque owing to the normal forces is obtained.

(2) $$P=\int_{0}^{R}dP=\int_{0}^{R}\left(1/2)\;\rho \;B\;{\left(U_{rel}\right)}^{2}\;[\;Cl\;sin\;\Phi -Cd\;cos\;\Phi ](crdr\right)$$

U_rel_ = release energy
P = power,
c = speed,
ρ = density
Φ= angle of attack during wind interaction
B = Blade Number
C_L_ = Coefficient of Lift
C_D_ = Coefficient of Drag

It is not the right strategy to increase the fatigue life of a blade by compromising on output; the proposed design of a turbine blade must have an identical surface area to the original one because the output of a blade directly depends on the projected area of a blade. By making the area of both designs comparable, this study alters the blade design to increase its fatigue life without compromising output. A projected surface area is used to adjust variables including the twist angle, blade length, and chord length.

**Figure 2:**
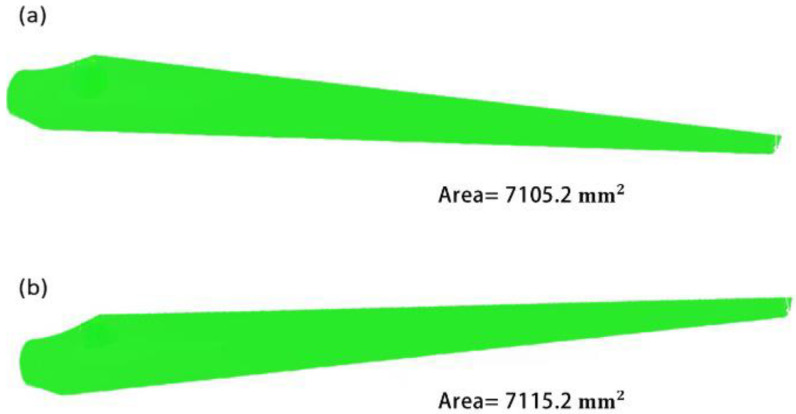
(**a**) Surface area of the original geometry of a turbine blade. (**b**) Surface area of a proposed geometry of a turbine blade.

Numerical simulation using SolidWorks is used to calculate the surface area of the two turbine blade designs (Figure 2). A 3D drawing is then created to connect the top and bottom faces of the turbine blade after it has been divided into 40 strips. The pressure that varies in those places is unreliable and varies due to turbulence because none of the portions are visible at the frontal plane confronting the wind. As a result, their area is not closely examined.

The finite element method is used to compute the engineering element’s structural and thermal analyses. The turbine blade is examined as a cantilever in the numerical examination because its length and cross-sectional area are much larger than its thickness. The program for numerical finite elements used to calculate numerous stresses is called ANSYS. A shell of 63 pieces with 4 nodes is used since the planar stress condition is anticipated. In both numerical and experimental tests, the blade’s substance was determined to be an aluminum alloy with Mechanical properties as shown in (Table 1).

**Figure 3:**
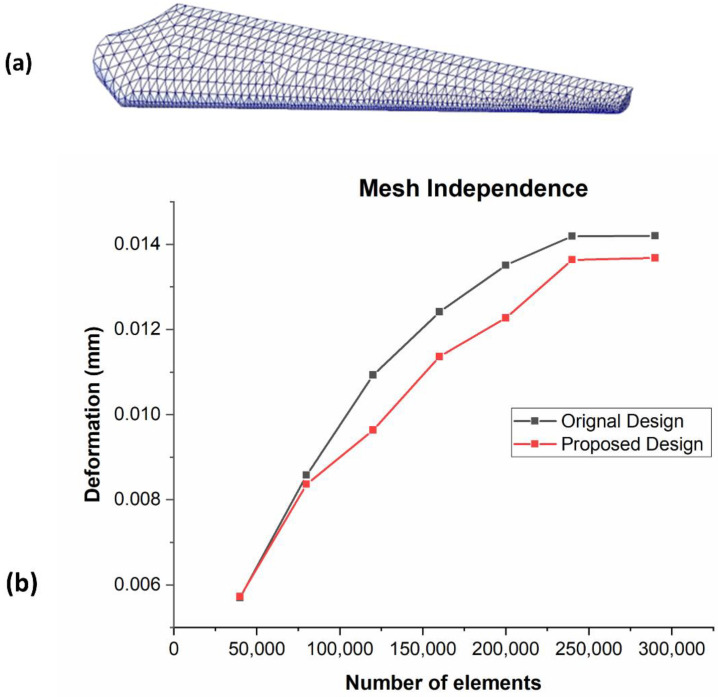
(**a**) Mesh representation of a turbine blade’s suggested geometry. (**b**) Mesh independence plot of the proposed and original turbine blade geometries.

When the aerodynamic normal forces, centrifugal forces, gravity force, and tangential forces are applied to the blade, the displacement of the blade does not change. The 16. 0 unstructured grid is initially used for the numerical inquiry; however, to increase precision, a sphere influence technique is used to congeal the grid around the model. Finding the ideal mesh and assuring the correctness of the outcomes both require mesh independence. As a result, the same technique described in these studies is used to perform the mesh independence test [23,24,25]. The mesh (Figure 3a) that was initially produced for the numerical investigation has a total of 2,143,020 components. The mesh is then modified, and consequently, there are 2,871,038 elements. It should be observed that 2,871,038 displays an ideal mesh when compared to a coarse mesh with 192,988 elements and 64,311 nodes. The computing time is accurately indicated on the best mesh and is at a minimum [7,26,27]. The grid dependence test is consistent with numerical simulations where the force coefficient for the finer mesh is 0.50 percent different from the coarse mesh (Figure 3b).

**Figure 4:**
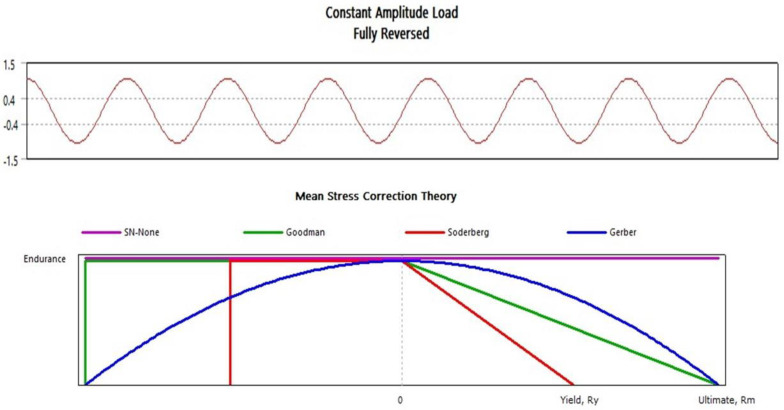
Goodman mean stress plot.

ANSYS is a numerical tool that calculates a blade’s fatigue damage using a variety of techniques, including stress-based, strain-based, and elastic displacement-based methods. The stress-based approach and the Goodman model (Figure 4) are used in this research work to replicate the numerical conditions with experimental ones. The Goodman model, which is based on the properties of the material, forecasts the fatigue life at a specified stress ratio (alternating and mean stresses) (ultimate strength and endurance strength).

In terms of blade length (L), chord length (C), and twist angle (Degrees), the finite element model and the applied aerodynamic loads are calculated. Since aluminum is a ductile material, the stresses in this research study are only evaluated for design purposes. They are applied to the beam element theory in accordance with the finite element theory. The rotor of a horizontal-axis wind turbine can be positioned upwind or downwind and is equipped with turbine blades (also known as airfoils). HAWTs can operate at high blade tip speeds and typically have two or three blades. The turbine blade operates at 1000 rpm in a real situation, with bearing pressure ranging from 6 to 9 bar and an ambient temperature of 55 °C. When compared to the blade’s spinning speed, the pressure load caused by air intake is incredibly minimal. As a result, the numerical analysis has a very compressed understanding of the size of a heat and pressure load.

**Figure 5:**
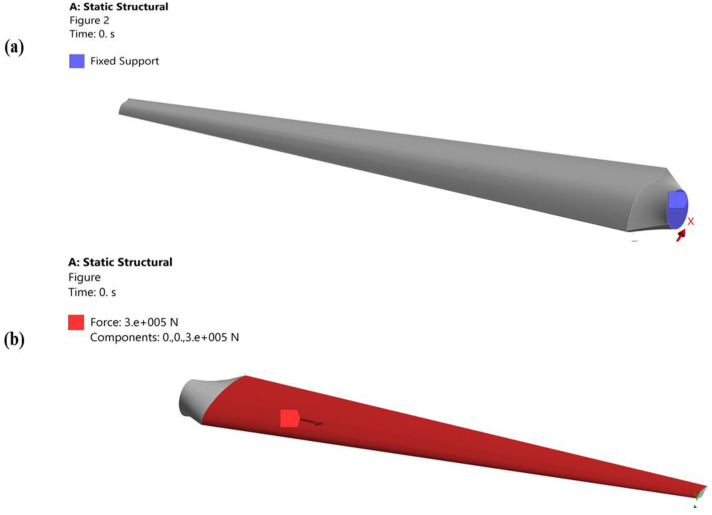
(**a**) Fixed support at the root of a turbine blade. (**b**) Petition of cyclic load along the length of a blade.

In ANSYS Workbench 22.0, numerical computation is carried out by fixing a blade’s root while applying stress throughout the blade’s length (Figure 5a,b). The centrifugal force is created by the rotational speed, which is applied as an angular velocity. Due to the simultaneous occurrence of deformations at the fan blade tip and blade root caused by rotational velocity, the blade root experiences increased stress in proportion to the numerical progression. The strain life technique is used in the numerical simulation as opposed to stress life analysis because it better depicts a fatigue action and considers localized plasticity that occurs prior to fracture initiation at the crucial area.

## 4. Results and Discussion

### SN Curve

**Figure 6:**
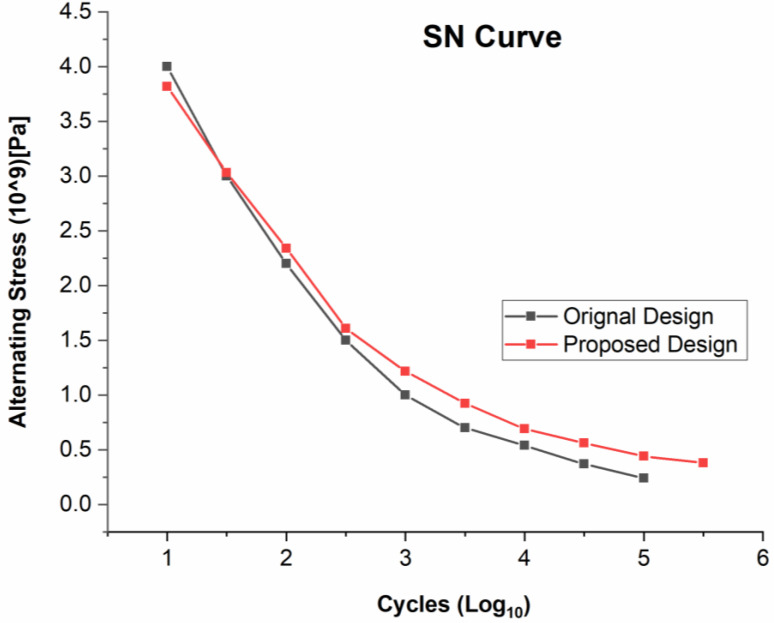
SN curve of both the geometries of a turbine blade.

The failure of turbine blades (both original and proposed) when a repetitive cyclic load is applied for a significant number of cycles is shown by the SN curve (Figure 6), which was determined below. The SN curve demonstrates that the turbine blade’s proposed design is more resilient and needs more stress cycles before failing due to resistance. Because each unit surface of the blade carries little stress that needs huge cycles to fail, the proposed shape increases the stress cycles for blade failure by dispersing the load evenly.

Static analysis of turbine blades is carried out by giving centrifugal bending load along its longitudinal axis at a temperature of 55 °C with pressure ranging from 6 bar to 9 bar.

**Figure 7:**
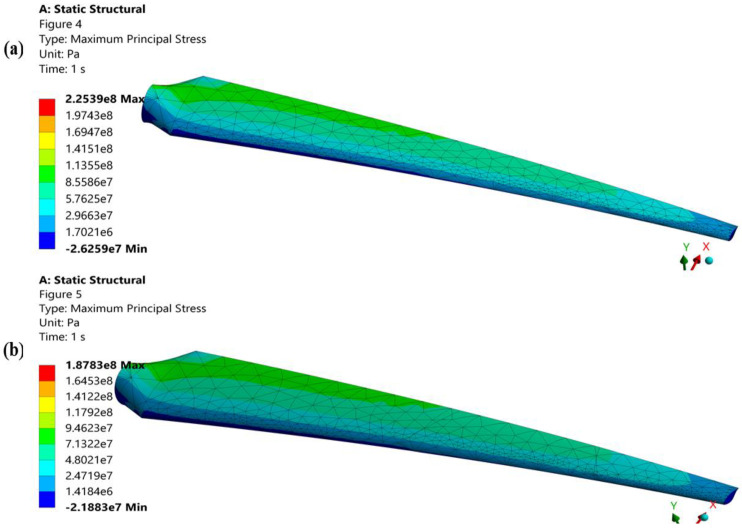
(**a**) Static structural stresses of the original geometry of a turbine blade. (**b**) Static structural stress of the proposed geometry of a turbine blade.

**Figure 8:**
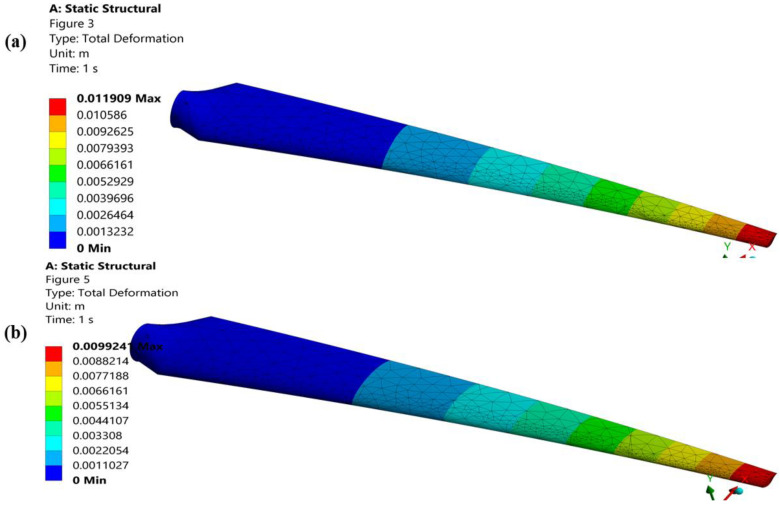
(**a**) Static structural deformation of the original geometry of a turbine blade. (**b**) Static structural deformation of the proposed geometry of a turbine blade.

According to the legends described above, (Figure 7), turbine blades fail due to low cycle fatigue because the repeated cyclic principal stresses in both blades are greater than the yield strength of a blade material, whereas the proposed geometry of a turbine blade makes the blade more stress resistant (Figure 7b), and as a result, less stress is generated along its axis. The blade’s proposed geometry has a thicker leading edge, which increases the blade’s penetrating surface area and reduces von Mises. In the early phases of fatigue life, plastic deformation controls the quick softening of a turbine blade and manifests the subsequent saturation up to fatigue failure. The plastic deformation of the blade determines the plastic strain that occurs in the plastic zone of the domain. The proposed geometry of a blade is shown in (Figure 8) to reduce the strain value by reducing plastic deformation in the numerical domain. Because of the proposed shape of a blade’s longer chord length, which slows down stress propagation, and compact blade length, which lessens overall deformation (Figure 8b), it has a slight plastic strain value. Based on deformation and produced stresses, the statistical operating system ANSYS calculates the fatigue life of a blade subjected to cyclic repeated load.

**Figure 9:**
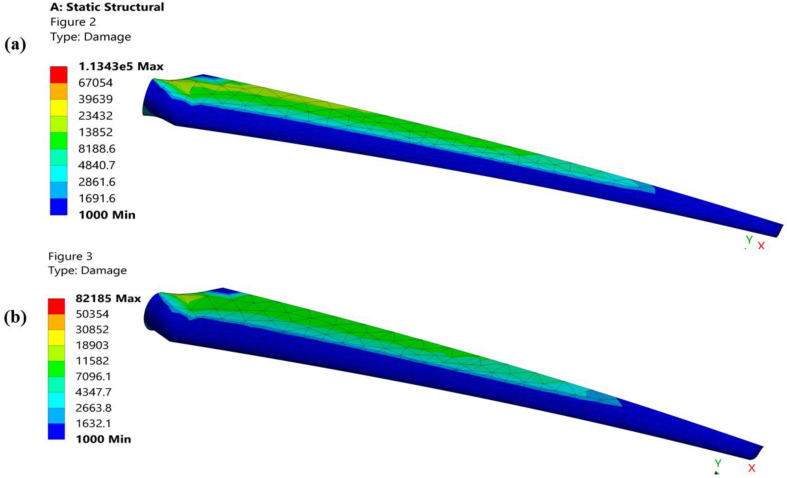
(**a**) Structural damage of the original geometry of a turbine blade. (**b**) Structural damage of the proposed geometry of a turbine blade.

The beginning and spread of a crack because of repeated cyclic pressures is what causes fatigue mutilation. Although the proposed geometry lessens the fatigue damage to the blade because of its enormous twist angle and chord length, Figure 9 shows damage to both geometries of a turbine blade. In a blade airfoil, the chord length minimizes the hollow space between the leading and trailing edges to minimize deformation while the twist angle minimizes stresses by increasing the instantaneous area.

**Figure 10:**
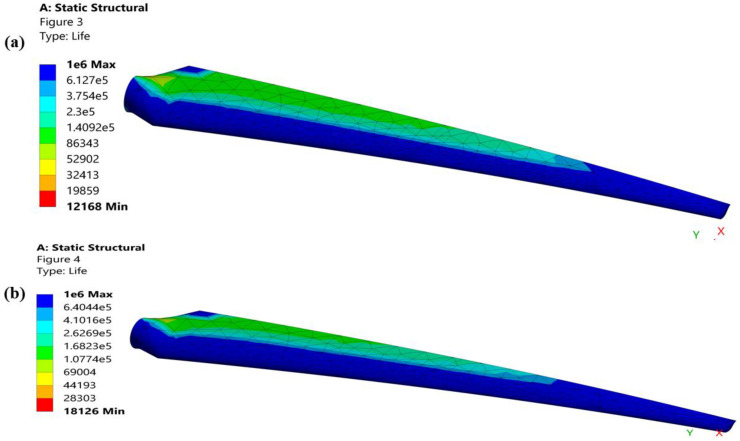
(**a**) Fatigue life of the original geometry of a turbine blade. (**b**) Fatigue life of the proposed geometry of a turbine blade.

The turbine blades’ maximum and minimum fatigue lives under the same conditions of cyclic repeated stress are shown in Figure 10. By reducing the dispersion of fatigue along the chord length, the suggested geometry increases the fatigue life of a turbine blade (Figure 10b). The chord length, blade length, and leading-edge thickness are ordered in the proposed design in a way that improves fatigue life. The chord length of a blade and the leading-edge thickness are directly correlated with fatigue life because a long chord length reduces stress and a thick leading edge minimizes the impact of a load by increasing the instantaneous area, whereas the fatigue life of a turbine is inversely correlated with the blade length because the blade length determines the proportion of deformation because of the proposed geometry.

## 5. Experimental Setup and Specimen Fabrication

**Figure 11:**
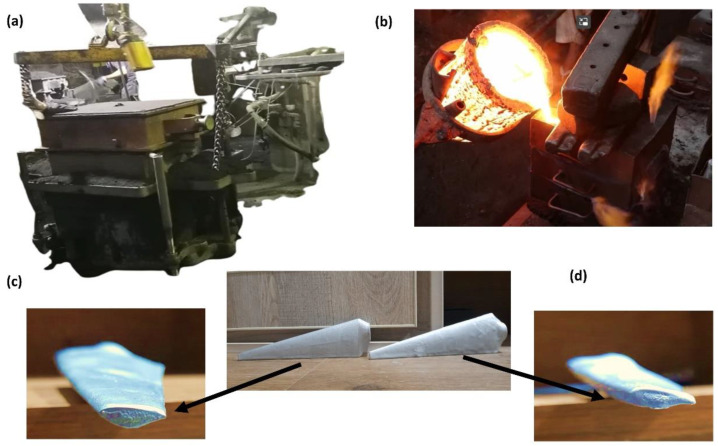
(**a**) Pneumatic mold constructer, (**b**) pouring of molten aluminum, (**c**) airfoils and prototypes of original design, and (**d**) airfoils and prototypes of a proposed design.

For the experimental investigation, prototypes of both (the original and proposed) design of turbine blades are fabricated by aluminum casting [28] (Figure 11). These parts are manufactured by making a sand mold and then pressurized by a pneumatic mold maker (Figure 11b) to acquire the desired shape. Molten aluminum is poured into the mold and cooled down slowly for uniform phase change. To improve the surface finish these are coated with epoxy resign and Aerodur (Figure 11c).

**Figure 12:**
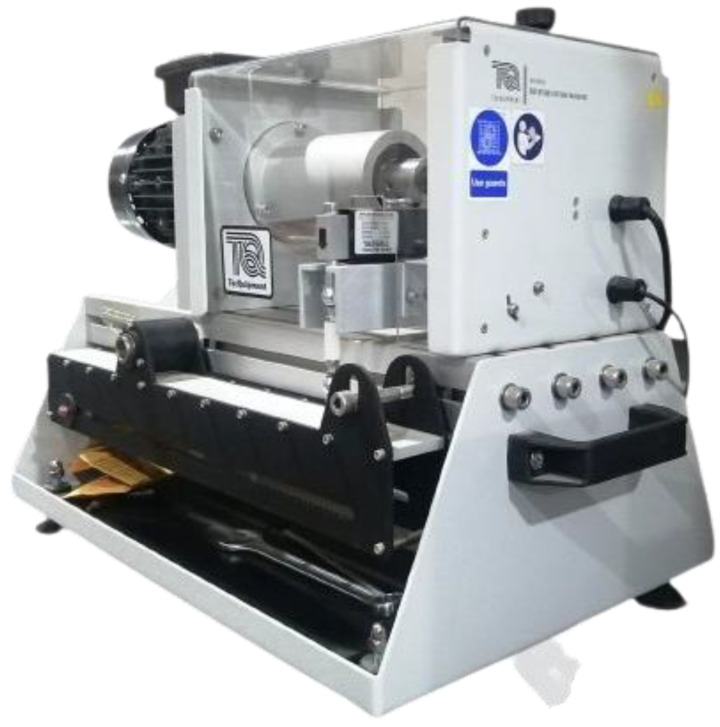
Low and high cycle fatigue machine (SM1090).

For experimental investigation of the fatigue life of turbine blades, an **SM1090V manufactured by Tecequipment** (Figure 12) fatigue testing machine is utilized. It is a bench-mounted machine for demonstrating the failure of materials when subjected to an alternating load and shows the outcomes of cyclic load for an immense number of cycles. **SM1090V** is a computer-operated machine equipped with VDAS software 20.0 [29] that computes the fatigue life of a specimen at low or high cycle cyclic stress.

## 6. Experimental Investigation

**Figure 13:**
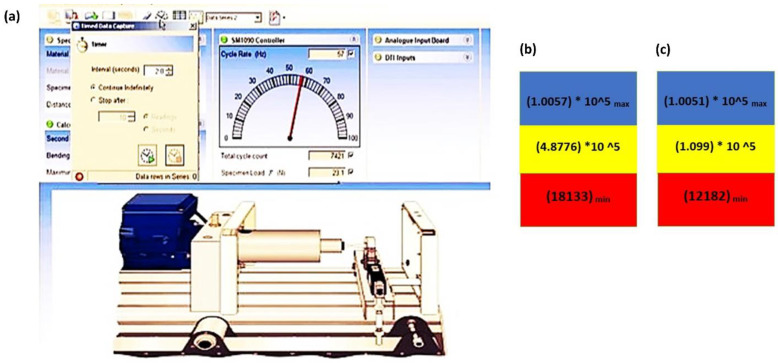
(**a**) Interface of SM1090 depicting the calibration of a cyclic load and Experimental fatigue life of proposed design (**b**) and original design (**c**) of a turbine blade.

A computer-controlled numerical machine called SM 1090 (Figure 12) computes the fatigue life for low and high-scale fatigue failure. To guarantee the accuracy of the results, it replicates every condition that is carried out during numerical simulations. The specimen is subjected to a steady cyclic load for an enormous number of cycles to calculate the fatigue life. The adaptation of a time interval and cycle count of stress on the interface of an SM1090 fatigue machine is shown in Figure 13a.

The experiment fatigue life of both horizontal-axis wind turbine geometries subjected to cyclic repetitive load is shown in Figure 13b, c. The efficiency and service or operation of a blade are mostly determined by the minimal fatigue life since the blade can cease functioning even in the early stages of fatigue. The suggested blade geometry increases fatigue life by reducing the fraction of deformation and sedating the stress propagation.

### Uncertainty Calculation

Although numerical results are shown to be reasonable like experimental data, these values have inherent uncertainty (Table 2). The first is that while boundary conditions are supposed to be constant in experimentation and constant in numerical analysis, they do not hold true in real-world circumstances. Second, the FL (EXP) and FL differences could be caused by adjustment faults in the SM1090 fatigue machine (SIM). Thirdly, the disparity between the experimental and numerical examinations may be due to adjustment effects in the SM1090 operating system (VDAS).

According to the findings, extending the blade fatigue life will improve the performance of turbine blades. By reducing fatigue damage (Figure 9) and raising the safety factor of fatigue examination, the proposed geometry of a blade increases fatigue resistance. Figure 7 shows that both designs fail due to fatigue failure because the cyclic pressures are greater than the blade material’s yield strength. By shortening the von Mises, the significant leading-edge thickness of a blade’s suggested geometry moderates fatigue failure. The blade root carries a significant load from the hub because it experiences the most deformation and the shortest fatigue life (Figure 10). Nevertheless, a proposed blade shape with a longer chord and a higher chamber strengthens the blade root by reducing both stress initiation and propagation.

## 7. Conclusions

In this study, the turbine blade underwent experimental and numerical fatigue testing. Through numerical and experimental testing, homogenous circumstances were used to assess parameters that are difficult to see in real-time. Attempts were also made to determine the variables that affect fatigue life and how they should be maximized. These are a few significant findings from the study:

1. In this research study, the operating system (VDAS) of the SM1090 (Figure 13) is calibrated with ANSYS, and the fatigue life is examined numerically and experimentally while the structure is being built;
2. Fatigue failure only happens when the cyclic stresses are greater than the blade material’s yield strength;
3. By slowing down the start of cyclic repeated stress, lengthening a blade’s chord immediately extends fatigue life;
4. Increasing the leading-edge thickness reduces cyclic stress since it represents the immediate area and lengthens the blade’s fatigue life;
5. Because the blade impacts the fraction of deformation, fatigue life relates inversely to blade length; therefore, blade length needs to be less to increase fatigue life;
6. The substantial hub stress causes the minimum fatigue life to occur in both blade geometries at the blade root. By extending an airfoil chamber, you can reduce the hub stress propagation and prolong the blade root life fatigue life;
7. The failure occurs at a low number of cycles governed by plastic deformation and related to cyclic loads, which is true for both turbine blade designs that experience fatigue.

## 8. Future Work

This research can be enhanced further by the following points in future works:

I. In the future, a turbine blade’s fatigue behavior will be examined numerically and experimentally, allowing for various points during the blade’s revolution to be analyzed and interpreted.
II. Within a predetermined framework, both the wind and the turbine blades are moving. Further tests may be carried out by using multiple direction loads to verify this numerically and empirically.

## Figures and Tables

**Table 1:** Mechanical properties of an aluminum alloy.

S.NO	Mechanical Properties	Value
**01**	Density	2.7 g/cm^3^
**02**	Ultimate Strength	990 Mpa
**03**	Yield Strength	240 Mpa
**04**	Modulus of elasticity	70 Gpa
**05**	Fatigue Strength	510 Mpa
**06**	Poisson Ratio	0.33
**07**	Melting Point	660 °C
**08**	Fatigue Limit range	0.06–0.1
**09**	Fatigue strength factor	01

**Table 2:** Uncertainty calculation of both the geometries of a turbine blade.

Original Design	Proposed Design
**Numerical Consideration**	**Numerical Consideration**
Minimum Fatigue life = F_L_ = 12,168	Minimum Fatigue life = F_L_ = 18,126
**Experimental Consideration**	**Experimental Consideration**
Minimum Fatigue life = F_L_ = 12,182	Minimum Fatigue life = F_L_ = 18,183
**Percentage Uncertainty**	**Percentage Uncertainty**
=(12,357 − 12,168)/(12,168) = 1.9%	=(18,490 − 18,126)/(18,126) = 3.5%

## Data Availability

Not applicable, all data already included in main file.
